# Ascorbic acid can promote the generation and expansion of neuroepithelial-like stem cells derived from hiPS/ES cells under chemically defined conditions through promoting collagen synthesis

**DOI:** 10.1186/s13287-020-02115-6

**Published:** 2021-01-09

**Authors:** Rui Bai, Yun Chang, Amina Saleem, Fujian Wu, Lei Tian, Siyao Zhang, Ya’nan Li, Shuhong Ma, Tao Dong, Tianwei Guo, Youxu Jiang, Yi You, Wen-Jing Lu, Hong Feng Jiang, Feng Lan

**Affiliations:** 1 Beijing Laboratory for Cardiovascular Precision Medicine, MOE Key Laboratory of Medical Engineering for Cardiovascular Diseases, MOE Key Laboratory of Remodeling-Related Cardiovascular Disease, Beijing Collaborative Innovation Center for Cardiovascular Disorders, Anzhen Hospital, Capital Medical University, Beijing, 100029 China; 2 Beijing Institute of Heart, Lung and Blood Vessel Diseases, Beijing, 100029 China; 3 Key Laboratory of Chinese Internal Medicine of Ministry of Education and Beijing, Dongzhimen Hospital Affiliated to Beijing University of Chinese Medicine, Beijing, China; 4 Center for Clinical Translation and Innovation, Peking University Shenzhen Graduate School, Shenzhen, 518055 China; 5 Shenzhen Bay Laboratory, Shenzhen, 518055 China; 6 Beijing Anzhen Hospital, Research Institute Building, Room 323, 2 Anzhen Road, Chaoyang District, Beijing, 100029 China; 7 State Key Laboratory of Cardiovascular Disease, National Center for Cardiovascular Diseases, Fuwai Hospital, Chinese Academy of Medical Sciences and Peking Union Medical College, Beijing, China; 8 Beijing Anzhen Hospital, Research Institute Building, Room 319, 2 Anzhen Road, Chaoyang District, Beijing, 100029 China

**Keywords:** Spinal cord injury, Neurospheres, Ascorbic acid, Long-term self-renewing neuroepithelial-like stem cells, Human pluripotent stem cells

## Abstract

**Introduction:**

Spinal cord injury (SCI) is a neurological, medically incurable disorder. Human pluripotent stem cells (hPSCs) have the potential to generate neural stem/progenitor cells (NS/PCs), which hold promise in the treatment of SCI by transplantation. In our study, we aimed to establish a chemically defined culture system using serum-free medium and ascorbic acid (AA) to generate and expand long-term self-renewing neuroepithelial-like stem cells (lt-NES cells) differentiated from hPSCs effectively and stably.

**Methods:**

We induced human embryonic stem cells (hESCs)/induced PSCs (iPSCs) to neurospheres using a newly established in vitro induction system. Moreover, lt-NES cells were derived from hESC/iPSC-neurospheres using two induction systems, i.e., conventional N2 medium with gelatin-coated plates (coated) and N2+AA medium without pre-coated plates (AA), and were characterized by reverse transcription polymerase chain reaction (RT-PCR) analysis and immunocytochemistry staining. Subsequently, lt-NES cells were induced to neurons. A microelectrode array (MEA) recording system was used to evaluate the functionality of the neurons differentiated from lt-NES cells. Finally, the mechanism underlying the induction of lt-NES cells by AA was explored through RNA-seq and the use of inhibitors.

**Results:**

HESCs/iPSCs were efficiently induced to neurospheres using a newly established induction system in vitro. lt-NES cells derived from hESC/iPSC-neurospheres using the two induction systems (coated vs. AA) both expressed the neural pluripotency-associated genes *PAX6*, *NESTIN*, *SOX1*, and *SOX2*. After long-term cultivation, we found that they both exhibited long-term expansion for more than a dozen generations while maintaining neuropluripotency. Moreover, the lt-NES cells retained the ability to differentiate into general functional neurons that express β-tubulin at high levels. We also demonstrated that AA promotes the generation and long-term expansion of lt-NES cells by promoting collagen synthesis via the MEK-ERK1/2 pathway.

**Conclusions:**

This new chemically defined culture system was stable and effective regarding the generation and culture of lt-NES cells induced from hESCs/iPSCs using serum-free medium combined with AA. The lt-NES cells induced under this culture system maintained their long-term expansion and neural pluripotency, with the potential to differentiate into functional neurons.

**Graphical abstract:**

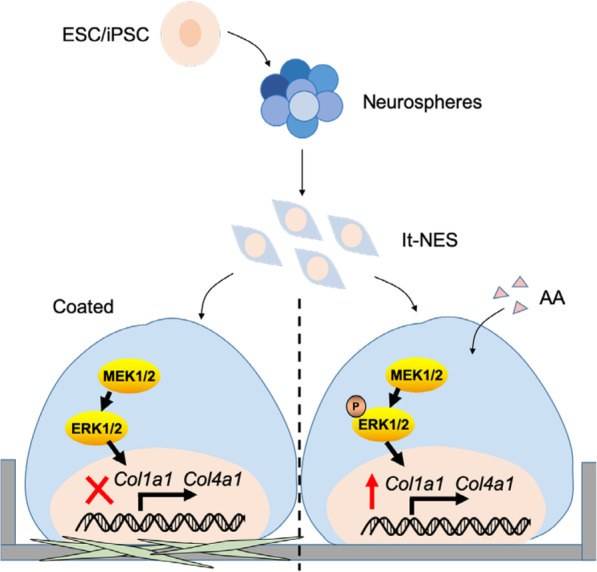

**Supplementary Information:**

The online version contains supplementary material available at 10.1186/s13287-020-02115-6.

## Introduction

Spinal cord injury (SCI) is a destructive neurodegenerative disorder with life-long consequences that often leads to irreversible changes because of the loss of neurons and glial cells [1, 2]. However, the quality of life of patients with SCI has been improved by the findings of past research; nevertheless, no effective therapy is currently available. In recent years, many experimental animal models have been generated to simulate human SCI, and the transplantation of neural stem/progenitor cells (NS/PCs) has been shown to be an effective treatment to cure neurological disorders and repair damaged brain tissue because of their ability to differentiate into neurons and glial cells and their competence to secrete neurotrophic factors [3, 4].

In the SCI research field, NS/PCs derived from mouse embryonic stem cells (mESCs) [5] and rat embryonic spinal cord [3] have been transplanted into the injured spinal cord of mice and rats, thus promoting functional recovery in animal models of SCI. Moreover, NS/PCs derived from the human fetal brain [6, 7] have been transplanted into injured spinal cord models in mice or nonhuman primates [6, 8], thus greatly promoting the development of stem-cell-based transplantation therapy for human patients. However, the collection of NS/PCs from the brains of aborted fetuses or surplus embryos is not allowed for clinical applications in Japan. This fact has been a major hindrance to the clinical use of human NS/PCs [9].

In this context, human embryonic stem cells (hESCs) [10, 11] and induced pluripotent stem cells (iPSCs), which were established by Yamanaka et al. [12, 13], have the ability to differentiate into NS/PCs, thus representing a rich source of neurons and glial cells [14]. Therefore, they provide a new approach for studying human neurodevelopment, for simulating neurological disease and for regenerative medicine. Moreover, iPSCs reprogrammed from somatic cells provide the new prospect of generating patient-specific cells for disease modeling and regenerative medicine [12]. The proliferation and differentiation abilities of iPSCs are almost equal to those of ESCs, and the use of iPSCs could circumvent the ethical issues and rejection in NS/PC transplantation related to the use of embryos and aborted tissues.

Neurospheres are a well-known culture system for NS/PC extension [15]. The neurosphere-induction protocols available currently for human pluripotent stem cells (hPSCs) depend on the formation of an embryoid body [16–18]; however, this method has many considerable disadvantages, such as unclear culture conditions, prolonged differentiation, and low differentiation efficiency. In addition to these methods, human long-term self-renewing neuroepithelial-like stem (lt-NES) cells [18] have reached the standard definition of NS/PCs, thus providing a more homogeneous and robust cell generation via monolayer adherent cultures [19]. Furthermore, all lt-NES cells derived from different hESCs/hiPSCs exhibit characteristics such as continuous expandability, stable neuronal and glial differentiation competence, and the capacity to generate functionally mature human neurons [14, 20, 21]. However, these lt-NES cell-culture systems require plates coated with adhesive materials, thus not being chemically clear culture systems. Therefore, the development of a culture system of lt-NES cells under chemically defined conditions is crucial.

Here, we describe for the first time a serum-free culture system to generate lt-NES cells from different hPSCs under chemically defined conditions without the need for pre-coating and avoiding the preparation of basement membranes, such as Matrigel matrix-coated, poly-l-lysine/laminin-coated, or inactivated MEF-feeder-coated plates [22, 23], with unclear chemical composition, which limit the clinical applications of NS/PCs. In this system, we applied ascorbic acid (AA), which promotes collagen synthesis through the MEK-ERK1/2 pathway [24], to induce the generation and long-term expansion of lt-NES cells. This method is expected to facilitate the clinical application of NS/PCs derived from hPSCs for the regenerative-medicine-based therapy of neurological disorders and injuries, such as SCI [25].

## Materials and methods

### Human PSC culture

Human PSCs including hESCs-H9 (H9, provided by WiCell Institute Inc., Madison, WI, USA) and urine-derived iPSCs (UiPSCs, provided by Cellapy: CA1002008, Beijing, China) were cultured in PSCeasy medium (Cellapy, China) on six-well plates (Corning, US) coated with a 1:500 dilution of hESC-matrigel (5 μg/cm^2^, Corning). Medium was changed every day. PSCs were passaged every 3–4 days at 70–80% confluence with 0.5 mM EDTA (Cellapy). All cells were maintained at 37 °C, 5% CO_2_ in incubator (Thermo Fisher Scientific, USA).

### Neurosphere differentiation of PSCs

**Fig. 1:**
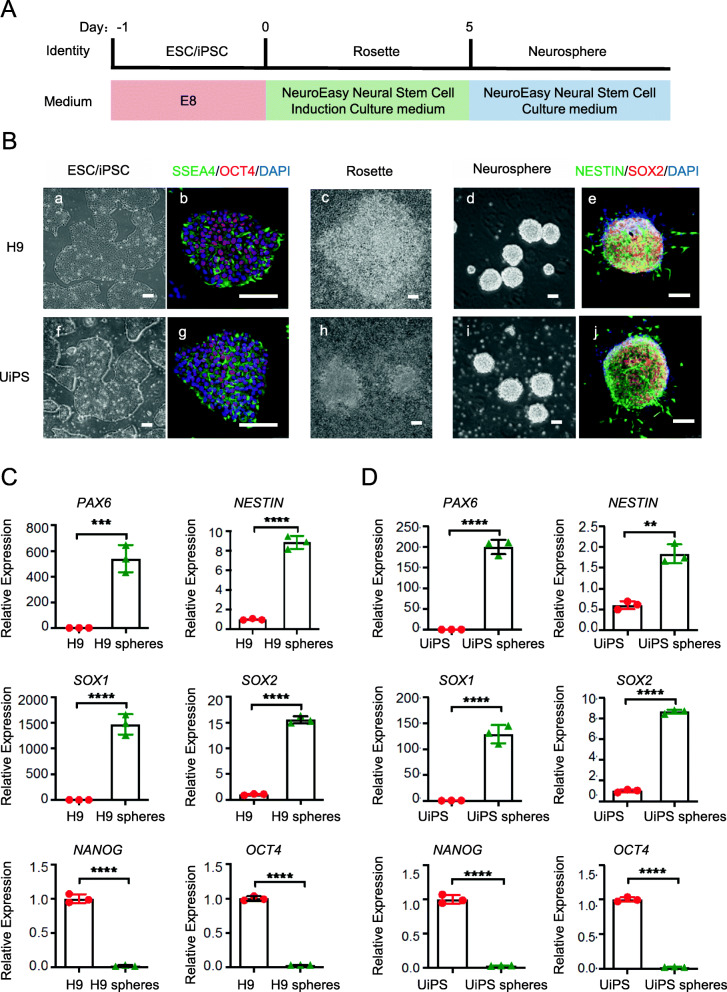
Generation of neurosphere from hiPS/ESCs. **a** The protocol for deriving neurospheres from H9 and UiPS. **b** The morphology and Immunostaining of H9 and UiPS at days − 1 (a, b, f, g), day 5 (c, h), and day 7 (d, e, i, j) during neural induction. Immunostaining showing emergence of SSEA4+ (green), OCT4+ (red) H9 and UiPS (b, g), and SOX2+ (red), NESTIN+ (green) neurospheres induced from H9 and UiPS at day 7 (e, j). Scale bars = 100 μm. Nuclei were counterstained with DAPI (blue). **c** Quantitative RT-PCR analysis of the expression level of neural progenitor markers *SOX1*, *PAX6*, *NESTIN*, and *SOX2* and pluripotency markers *NANOG* and *OCT4* of H9 and H9-neurospheres. **d** Quantitative RT-PCR analysis of the expression level of neural progenitor markers *SOX1*, *PAX6*, *NESTIN*, and *SOX2* and pluripotency markers *NANOG* and *OCT4* of UiPS and UiPS-neurospheres. *n* = 3 each. Data are expressed as means ± SD.*P < 0.05, **P < 0.01 vs. control

The protocol of neurosphere differentiation was shown in Fig. 1A. When hPSCs were grown for 4 days on human recombinant laminin fragment iMatrix-511 (Nippi, Japan)-coated (0.5 μg/cm^2^) 6-well plate, at which time they reached 100% confluence, namely at day 0 of induction of differentiation, cells were washed with PBS (Hyclone, USA), and then added the 2 ml NeuroEasy Human Neural Stem Cell Induction Culture medium (Cellapy, DMEM/F12 containing 500 μg/mL recombinant human albumin (A0237, Sigma-Aldrich)+2 μM Chir99021(Selleck, China)) per well, and change the medium every 2 days until the cells exhibited a rosette morphology (5–6 days). And then, cells were dissociated with Accutase (Innovative Cell Technologies, USA) at 37 °C for 10 min and resuspended by NeuroEasy Human Neural Stem Cell Culture medium (Cellapy, DMEM/F12 containing 2% NeuroEasy Human Neural Stem Cell supplement (xeno-free version of B27, Cellapy), supplemented with 20 ng/ml FGF2, 25 ng/ml EGF, and 25 ng/ml heparin) on low cell-adhesion plates. A ROCK inhibitor, Y27632 (Selleck, China) at a concentration of 10 μM, was used only at the time of plating. Next day, cell aggregated to form neurospheres, and the medium was changed to neural stem cell culture medium without Y27632. Neurospheres were passaged every 3–4 days when they grow to 200 μm in size with Accutase. Medium was changed every 2–3 days. All cells were maintained at 37 °C, 5% CO_2_ in incubator.

### lt-NES cell differentiation of neurosphere

When the neurospheres grow to 200 μm in size, we perform PBS washing and dissociated neurospheres with Accutase at 37 °C for 10 min and resuspended by lt-NES cells culture medium (DMEM/F12 containing 2% N2 (xeno-free version, Cellapy), supplemented with 20 ng/ml FGF2, 25 ng/ml EGF and 25 ng/ml heparin) on gelatin-coated plate. When we identified the effect of AA in the generation and expansion of lt-NES, uncoated-plates were used and 50 μg/ml L-ascorbic acid-2-phosphate (Selleck) was added to the lt-NES cells culture medium during resuspension, long-term culture, and other experimental procedures.

### Cell viability and proliferation assays

Cell viability and proliferation was measured by the Cell Counting Kit-8 (CCK-8) assay (Dojindo, Japan). The lt-NES cells under different condition were plated in 96-well plates (3000 cells/well), and cell viability was detected after 12 h according to the manufacturer’s protocol. Cell proliferation was detected every 24 h. At every point in time, 10 μl of CCK-8 reagent was added to each well, and the plate was incubated for 2 h at 37 °C. And then, each sample was measured absorbance at 450 nm using an automatic microplate reader (BioTek Synergn 4, USA). All experiments had three technical replicates each.

### Differentiation of lt-NES cells into generic neurons and glia

lt-NES cells were dissociated by for 10 min and plated on human recombinant laminin fragment iMatrix-511-coated 12-well plate with glass slides at a low density of 2 × 10^4^ cells/cm^2^ in neuron maintenance medium (DMEM/F12 containing 2% NeuroEasy Human Neural Stem Cell supplement (xeno-free version of B27, Cellapy)), and half of the medium was changed every 2 or 3 days.

### RNA extraction and quantitative real-time PCR

Cells were lysed and harvested using TRIzol™ Reagent (Invitrogen, USA) according to the manufacturer’s instructions. RNA samples were treated with DNase I (RNase free) (Beyotime, China), RNA was quantified with NANO drop 2000 (Thermo Fisher Scientific), and 1 μg RNA was reverse transcribed into cDNA using the PrimeScript™ RT Master Mix reverse transcription System (TaKaRa, Japan). The levels of relative gene expression were analyzed by quantitative reverse transcriptase PCR (qRT-PCR) with TB Green™ Premix Ex Taq™ II (TaKaRa) using the iCycler iQ5 (Bio-Rad, USA). The housekeeping gene GAPDH was used for internal normalization and the relative quantification of gene expression was calculated according to the △CT method. The qRT-PCR primers are listed in Supplementary Table S1.

### RNA-seq processing and biological information analysis

Clustering and sequencing were executed by Novogene Corporation (Tianjing, China). The clustering of the index-coded samples was performed on a cBot Cluster Generation System using TruSeq PE Cluster Kit v3-cBot-HS (Illumia). After cluster generation, the library preparations were sequenced on an Illumina Hiseq platform. Raw data (raw reads) of fastq format were processed through in-house perl scripts. All the downstream analyses were based on the clean data with high quality. Differential expression analysis was performed using the DESeq2 R package (1.16.1). The resulting *P* values were adjusted using the Benjamini and Hochberg’s approach for controlling the false discovery rate. Genes with an adjusted *P* value < 0.05 found by DESeq2 were assigned as differentially expressed. Gene Ontology (GO) enrichment analysis of differentially expressed genes was implemented by the clusterProfiler R package, in which gene length bias was corrected. GO terms with corrected *P* value less than 0.05 were considered significantly enriched by differential expressed genes. KEGG is a database resource for understanding high-level functions and utilities of the biological system from molecular-level information. We used clusterProfiler R package to test the statistical enrichment of differential expression genes in KEGG pathways.

### Immunofluorescence staining

Cells were fixed with 4% PFA (Solarbio, China) for 10–15 min at room temperature, permeabilized with 0.3% Triton X-100 (Sigma) for 10–15 min at room temperature, blocked with 3% bovine serum albumin (Solarbio) for 45–60 min at room temperature, and then incubated with primary antibodies against OCT4 (1:100; Santa Cruz Biotechnology), SSEA4 (1:100; Santa Cruz Biotechnology), SOX2 (1:100; Santa Cruz Biotechnology), NESTIN (1:100; Sigma), β3-tubulin (1:100; Abcam), and GFAP (1:100; Abcam) overnight at 4 °C. And then cells were incubated with secondary antibodies: Goat anti-Rabbit IgG Alexa Fluor 488 (1:200; Invitrogen) and Goat anti-Mouse IgG Alexa Fluor 594 (1:200; Invitrogen) for 1 h at 37 °C. Wash with PBS three times before each step. Nuclei were stained with DAPI (300 nM, Invitrogen) for 15 min at room temperature. Fluorescence images were captured by Leica DMI 4000B fluorescence microscope and Leica TCS SP5 MP confocal laser scanning microscope (Leica, Germany).

### Western blotting

Cells were lysed using tissue protein extraction reagent (Thermo, USA) containing phosphatase inhibitor cocktail (1:100, Thermo), protease inhibitor cocktail (1:100, Thermo), and 5 mM EDTA (Thermo); lysates were oscillated and centrifuged (13,000×*g*, 15 min, 4 °C). The supernatant was collected and stored at − 80 °C. The protein concentrations were determined using the BCA protein assay kit (Thermo). The protein was mixed with 5X SDS-PAGE protein loading buffer (Beyotime, China) and denatured by 100 °C water bath. Then, the samples denatured were electrophoresed in 10% SDS-PAGE and transferred to PVDF membranes using transfer device (Bio-Rad). The membranes were blocked with 5% non-fat milk prepared in TBST for 1 h at 37 °C and then incubated at 4 °C overnight with the primary antibodies: ERK1/2 (1:1000, Cell Signaling Technology, p44/42 MAPK Rabbit mAb, 4695), p-ERK1/2 (1:1000, Cell Signaling Technology, Phospho-p44/42 MAPK (Erk1/2) XP®, Rabbit mAb, 4370), and the internal normalization mouse anti-GAPDH (1:1000, Santa Cruz Biotechnology). Next, the membranes were washed in TBST, incubated with secondary antibody: Goat anti-Rabbit IgG (H + L) IRDye 800CW or Goat anti-Rabbit IgG (H + L) IRDye 800CW (1:20,000; LI-COR) for 1 h at 37 °C. The images were observed with a UVA Bio Imaging System and analyzed with ImageJ software.

### Data analysis and statistics

All data are analyzed by means ± standard errors of the means (S.E.M.). Statistical significance was evaluated using two-sided *t* test for two groups and using one-way ANOVA test for statistical differences of multiple groups. Significant differences were considered when *P < 0.05, **P < 0.01.

## Results

### Generation of neurospheres from hESCs/iPSCs

To evaluate the applicability of our approach, studies were performed in parallel in two cell lines, i.e., hESC-H9 and hiPSCs, produced from urine cells [26] via standard Sendai Reprogramming. Urine-iPSCs (UiPSs) were morphologically identical to hESC-H9 and expressed representative pluripotent markers, including SSEA4 and OCT4 (Fig. 1B (a, b, f, g)).

Neurospheres for culturing NS/PCs in vitro have been reported and are widely used [16]. The protocol used for neurosphere induction from hESC/hiPSCs is shown in Fig. 1A. HESC/hiPSCs can be efficiently differentiated to neurospheres with rosette formation using the neural stem cell induction medium, and rosette dissociation can be achieved using the neural stem cell medium. In the process of differentiation, the morphology of the transition from the hPSC to the neurosphere phenotype is shown in Fig. 1B. Significant changes in cell morphology accompanied cell proliferation. At the hPSC stage, cells had expanded and displayed a round, colony morphology (Fig. 1B (a, b, f, g)). On day 5 of neural induction, cells exhibited a rosette morphology (Fig. 1B (c, h)). On day 6, cells were digested and resuspended to form neurospheres (Fig. 1B (d, i)). By starting with pluripotent stem cells at 100% confluence, it is possible to obtain 20–40-fold increases in the number of NS/PCs obtained within 6 days; thus, it is possible to generate a quantity of NSCs that is sufficient to meet the needs of clinical applications.

The relative gene-expression analysis shown in Fig. 1C confirmed the identity of the neurospheres at day 7 through the progressive loss of hPSC pluripotency markers, such as *NANOG* and *OCT4*, and a significant increase in the expression of the neural progenitor markers *SOX1*, *PAX6*, *NESTIN*, and *SOX2*, which define early neural fate. At day 7 of neural induction, neurospheres were plated for immunostaining with pluripotent NS/PC markers; most of the cells were positive for the NS/PC markers SOX2 and NESTIN (Fig. 1B (e, j)). Neurosphere generation and gene expression were evaluated using two cell lines, H9 and UiPS, with no significant differences observed between them (Fig. 1C, D).

These results suggest that neurospheres derived from hESCs/hiPSCs using neural induction medium possess the NS/PC phenotype.

### AA promoted the generation of lt-NES cells from neurospheres under chemically defined conditions

Although neurospheres exhibit a heterogeneous character and tent to reproduce the neural development process, monolayer adherent cultures provide a more robust and homogeneous cell-generation system [19].

**Fig. 2:**
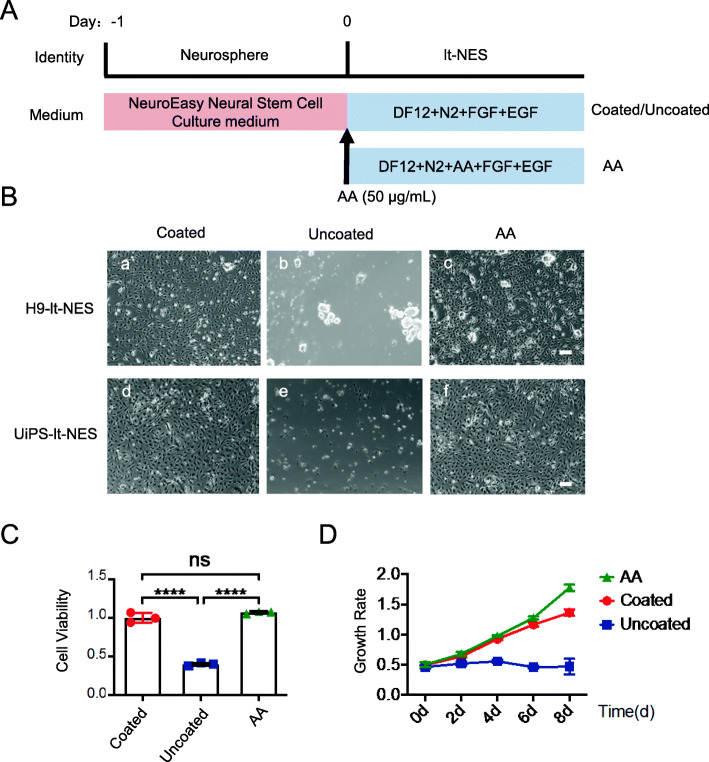
lt-NES cells can be generated from H9/UiPS-neurospheres under a chemically defined culture system using N2+AA medium. **a** The lt-NES cells derivation protocol from neurospheres. **b** The morphologies of the lt-NES cells in N2 medium (coated/uncoated) and N2+AA (AA). Scale bars = 100 μm. **c** The viability of P0 lt-NES cells was assessed by a CCK-8 assay after plated for 12 h. **d** Expansion capacity of lt-NES cells investigated by continuous culture in N2 medium (coated/uncoated) and N2+AA (AA) in 8 days. *n* = 3 each

We have applied many protocols to obtain lt-NES cells (Figure S1) and found that AA can promote the stable attachment of lt-NES cells. To evaluate the efficiency of using AA to induce lt-NES cells, we subsequently performed paralleled derivation of lt-NES cells from H9/UiPS-neurospheres using conventional N2 medium with gelatin-coated plates and N2+AA medium without gelatin-coated plates. The lt-NES cell-derivation protocol from neurospheres is shown in Fig. 2A. Neurospheres were dissociated into single cells and plated onto gelatin-coated/uncoated plates containing the N2 medium with FGF2 and epidermal growth factor (EGF) (referred to as coated/uncoated hereafter) or onto uncoated plates containing the N2+AA medium with FGF2 and EGF (referred to as AA hereafter). The morphologies of the lt-NES cells derived from H9/UiPS-neurospheres in N2 medium (coated) and N2+AA medium (AA) were comparable with each other (Fig. 2B (a, c vs. d, f)), with few lt-NES cells detected in uncoated plates (Fig. 2B (b, e)). The viability of lt-NES cells in the different culture conditions was evaluated by a CCK-8 assay (Fig. 2C), which revealed no differences in the cell viability of the lt-NES cells in coated and AA conditions at 12 after plating. Growth kinetics showed no significant differences in the proliferation of the lt-NES cells in the coated and AA conditions (Fig. 2D), which suggests that lt-NES cells expanded in the N2+AA medium (AA) into a large number of cells, with an expansion of almost three-fold observed upon each passage (lt-NES cells were passaged twice a week on average).

**Fig. 3:**
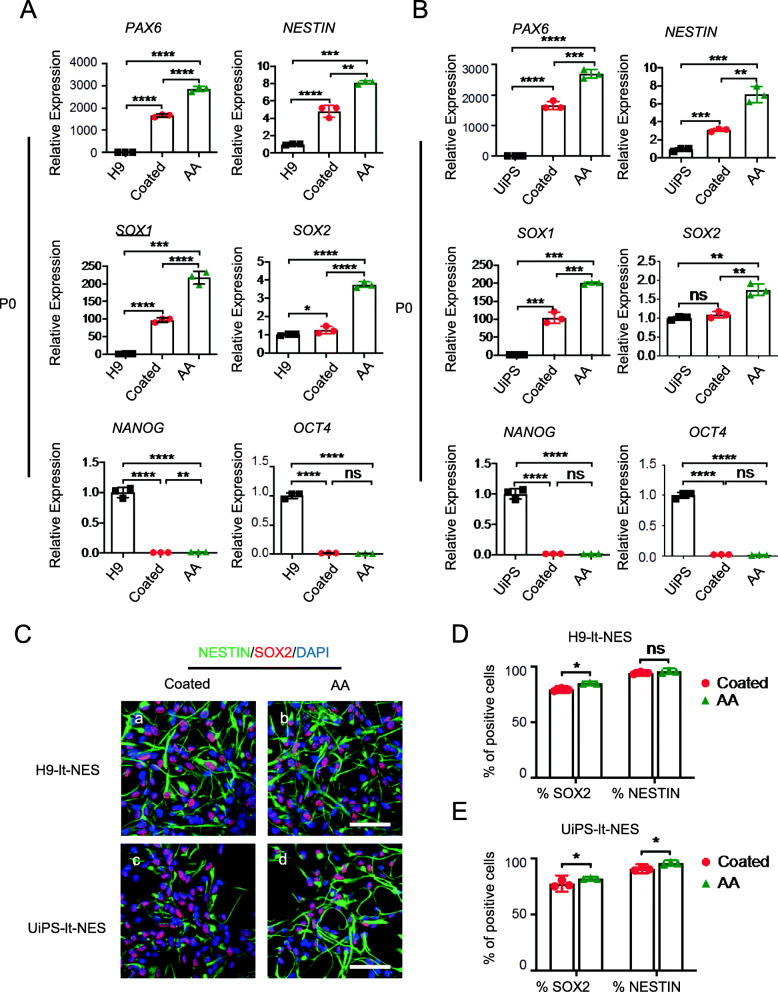
N2+AA medium can maintain the neural pluripotency of lt-NES cells. **a** Quantitative RT-PCR analysis of the expression level of NSC markers *SOX1*, *PAX6*, *NESTIN*, and *SOX2* and pluripotency markers *NANOG* and *OCT4* of P0 H9 neurosphere-derived lt-NES cells. **b** Quantitative RT-PCR analysis of the expression level of NSC markers *SOX1*, *PAX6*, *NESTIN*, and *SOX2* and pluripotency markers *NANOG* and *OCT4* of P0 UiPS neurosphere-derived lt-NES cells. *n* = 3 each. Data are expressed as means ± SD.*P < 0.05, **P < 0.01 vs. control. **c** Immunofluorescence for NSC markers SOX2 (red) and NESTIN (green) of P0 H9 & UiPS neurosphere-derived lt-NES cells. Scale bars = 100 μm. Nuclei were counterstained with DAPI (blue). **d** Quantification of SOX2- and NESTIN-positive cells of immunofluorescence of P0 H9 neurosphere-derived lt-NES cells. P, passage. **e** Quantification of SOX2- and NESTIN-positive cells of immunofluorescence of P0 UiPS neurosphere-derived lt-NES cells

All cells cultured using the two methods expressed the NS/PC markers *SOX1*, *PAX6*, *NESTIN*, and *SOX2* (Fig. 3A, B) and exhibited low expression of the hPSC pluripotency markers *NANOG* and *OCT4* in passage one. Of note, N2+AA (AA) cultured cells exhibited higher expression levels of NS/PC markers than did N2 cultured (coated) cells, which indicates that AA can promote the neural pluripotency of lt-NES cells. The immunocytochemical analysis indicated the absence of significant differences in the percentage of NS/PC marker-positive cells (Fig. 3C–E); however, there were slightly more SOX2-positive cells in the N2+AA culture, indicating that N2+AA is the optimal culture condition.

These data suggest that N2+AA medium in plates without gelatin coating is suitable for the induction and growth of lt-NES cells.

### AA can maintain the long-term expansion and neural pluripotency of lt-NES cells

**Fig. 4:**
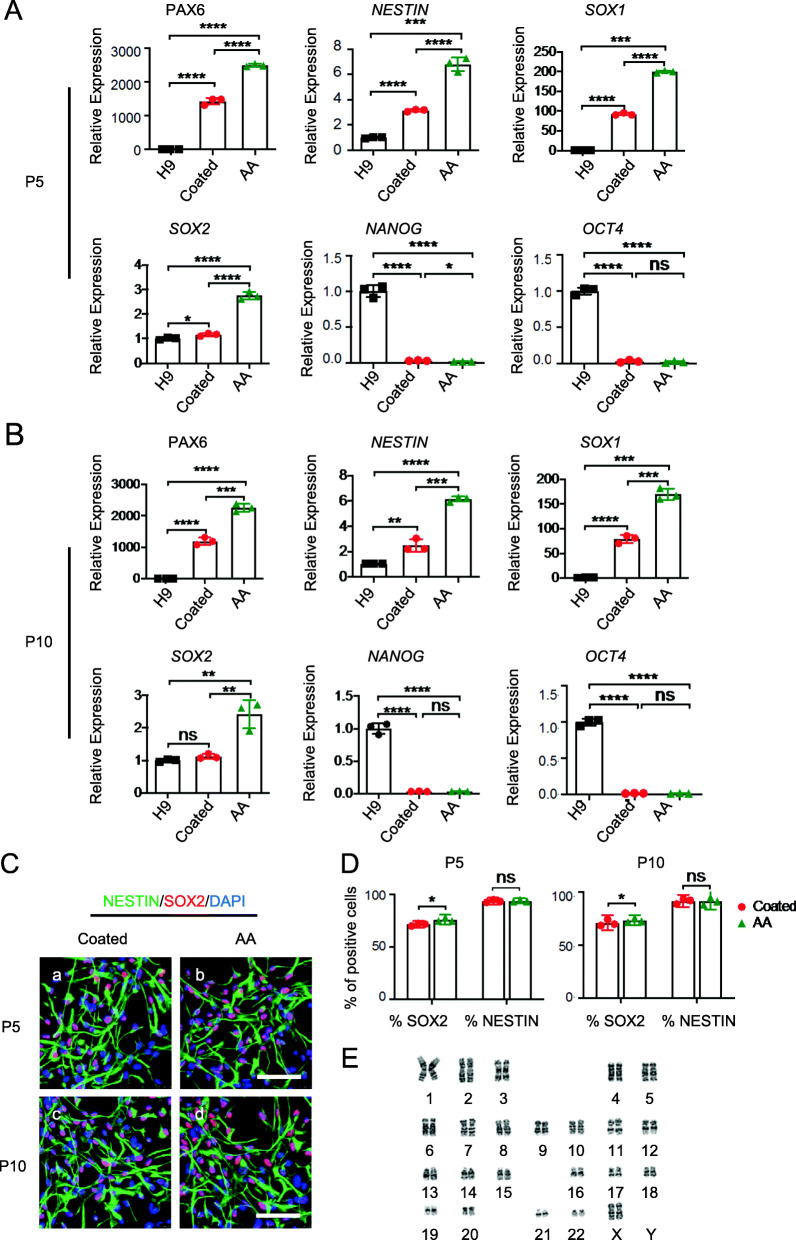
lt-NES cells can maintain long-term expansion and neural pluripotency using N2+AA medium. **a** Quantitative RT-PCR analysis of the expression level of NSC markers *SOX1*, *PAX6*, *NESTIN*, and *SOX2* and pluripotency markers *NANOG* and *OCT4* of P5 H9 neurosphere-derived lt-NES cells. **b** Quantitative RT-PCR analysis of the expression level of NSC markers *SOX1*, *PAX6*, *NESTIN*, and *SOX2* and pluripotency markers *NANOG* and *OCT4* of P10 H9 neurosphere-derived lt-NES cells. *n* = 3 each. Data are expressed as means ± SD.*P < 0.05, **P < 0.01 vs. control. **c** Immunofluorescence for NSC markers SOX2 (red) and NESTIN (green) of P5&P10 H9 neurosphere-derived lt-NES cells. Scale bars = 100 μm. Nuclei were counterstained with DAPI (blue). **d** Quantification of SOX2+ and NESTIN+ cells of immunofluorescence. P, passage. **e** Representative karyotypes of H9 neurosphere-derived lt-NES cells at passage 15

To verify whether the N2+AA culture system supports excellent proliferation and preserves the neural pluripotency of lt-NES cells, we cultured the cells in N2 (coated) and N2+AA (AA) conditions to 15 generations and identified the capacity of proliferation and the potential of neural differentiation by qPCR and immunocytochemistry. First, we counted the number of cells in each generation to calculate the cumulative population doubling and describe the expansion capacity of lt-NES cells in long-term culture. As shown in Figure S2, lt-NES cells grown in both coated and AA conditions could be cultured for 15 generations and exhibited vigorous growth, with similar growth rates. Next, the relative gene-expression analysis by qPCR shown in Fig. 4A, B showed that cells cultured in N2 (coated) or N2+AA (AA) medium to passage 5 and passage 10 expressed the NSC markers *SOX1*, *PAX6*, *NESTIN*, and *SOX2* and had almost no expression of the pluripotency markers *NANOG* and *OCT4*. Moreover, analyses of expanded lt-NES cells by immunofluorescence staining showed consistent expression of NSC markers, including SOX2 (> 75%) and NESTIN (> 95%) at passage 5 and passage 10 (Fig. 4C, D). Furthermore, the N2+AA medium was able to maintain a normal karyotype over 15 passages (Fig. 4E).

The results reported above revealed that N2+AA without gelatin coating can maintain the long-term expansion, yield excellent proliferation, and preserve the neural pluripotency of lt-NES cells.

### lt-NES cells cultured in N2+AA can differentiate into functional neurons

**Fig. 5:**
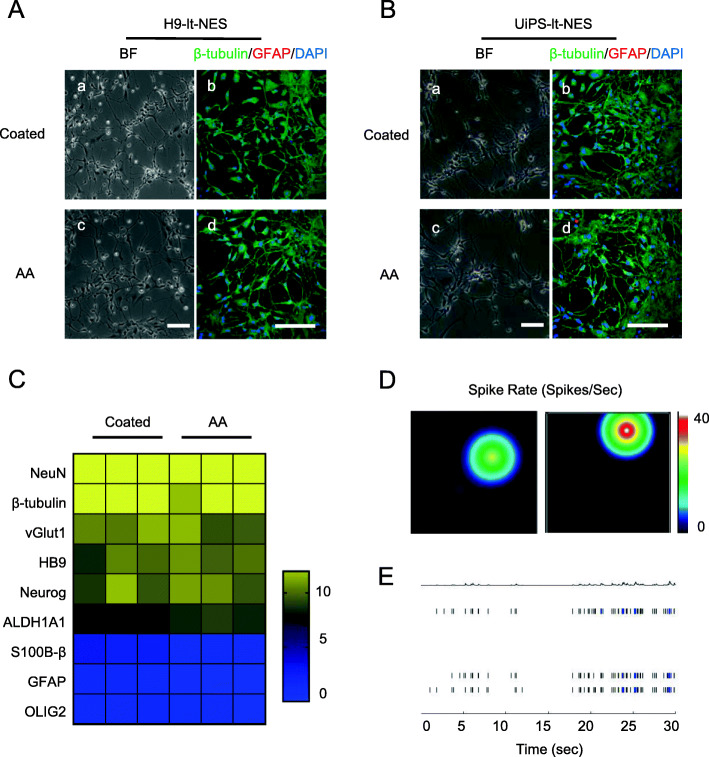
lt-NES cells differentiated into functional neurons. **A** Neurons and glia differentiated from P10 H9 neurosphere-derived lt-NES cells (a, c). Immunofluorescence for neuronal marker class III β-tubulin (green) and glia marker GFAP (red) of Neurons and glia (b, d). **B** Neurons and glia differentiated of P10 UiPS neurosphere-derived lt-NES cells (a, c). Immunofluorescence for neurons marker class III β-tubulin (green) and glia marker GFAP (red) of neurons and glia (b, d). Scale bars = 100 μm. Nuclei were counterstained with DAPI (blue). **C** Quantitative RT-PCR analysis of the expression level of neurons markers NeuN, Neurog, β-tubulin, the glutamatergic neuron marker vGlut1, the motor neuron marker HB9 and the dopaminergic neuron marker ALDH1A1, astrocyte marker GFAP, and the oligodendrocyte marker OLIG2. Transcript levels were normalized to undifferentiated lt-NES cells, *n* = 3 each. **D** Representative images of activity map of neurons differentiated from H9 neurosphere-derived lt-NES cells on the 24-electrode array. **E** Spikes of neurons differentiated from H9 neurosphere-derived lt-NES cells in 30 s

The neurons generated from NSCs provide a useful model for studying human diseases, drug screening, toxicity testing, and cell therapy. To test the neuronal differentiation potential of lt-NES cells cultured in N2+AA medium, we assessed whether the lt-NES cells could differentiate into generic neurons and glial. As shown in Fig. 5A (a, c) after 2 weeks of neuronal differentiation, H9-lt-NES cells in the coated and AA culture systems differentiated into a large number of neurons using neuron maintenance medium. Moreover, this phenomenon could be reproduced in UiPS-lt-NES cells, as they were also able to differentiate into a substantial number of neurons (Fig. 5B). As demonstrated by the immunocytochemical analysis of the neuronal marker class III β-tubulin and the glia marker glial fibrillary acidic protein (GFAP) (Fig. 5A (b, d)), the neurons produced by our system were highly pure (≥ 95% class III β-tubulin-positive neurons; < 5% GFAP-positive glia) and could be maintained in long-term culture. The quantitative RT-PCR analysis showed that neurons generated via the neuronal differentiation of N2 (coated) and N2+AA (AA) cultured lt-NES cells exhibited similar gene-expression patterns (Fig. 5C): after 14 days in the neuronal culture medium, the expression of neuronal type markers *NeuN*, *Neurog2*, and *β-tubulin*, the glutamatergic neuron marker *vGlut1*, the motor neuron marker *HB9*, and the dopaminergic neuron marker *ALDH1A1* was upregulated, whereas the expression of the astrocyte markers *S100B-β* and *GFAP* and the oligodendrocyte marker *OLIG2* was low. The use of a neuronal culture medium that is conducive to the growth of glial cells (such as adding thyroxine (T3) and large amounts of insulin) enhanced the expression of the astrocyte marker *GFAP* and the oligodendrocyte marker *OLIG2* (Figure S3). Subsequently, we investigated the electrophysiological activity of neurons differentiated from H9-lt-NES cells using a microelectrode array (MEA) recording system. We plated lt-NES cell-derived neurons onto the MEA plate, followed by their culture in the neuronal maintenance medium and the recording of their spontaneous activity (Fig. 5D). The spontaneous activity of the lt-NES cell-derived neurons gradually increased and was maintained at a high level for 50 days after differentiation (Fig. 5E). This indicates that N2+AA-cultured lt-NES cell-derived neurons were functional and able to maintain long-term spontaneous neuronal activity.

These data indicate that N2+AA-cultured lt-NES cells have the capacity to differentiate into generic functional neurons and glial.

### Transcriptome analysis of lt-NES cells

**Fig. 6:**
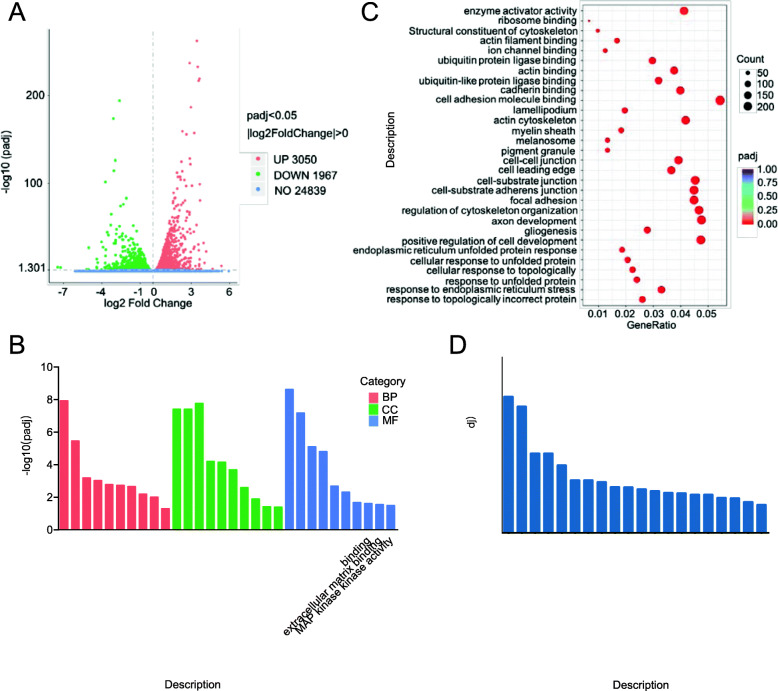
Transcriptome analysis of lt-NES cells cultured by coated or AA. **a** Volcano plot of upregulated (red) or downregulated (green) transcripts in AA cultured lt-NES cells vs. coated cultured. **b** Significantly enriched Gene Ontology (GO) terms in AA cultured lt-NES cells. **c** GO terms categorization and distribution of differentially expressed genes. GO terms were processed and categorized under three main categories (biological process, cellular component, and molecular function). **d** KEGG (Kyoto Encyclopedia Genes and Genomes) pathway rich detail

To explore the changes in gene expression, functional consequences, and potential molecular mechanism caused by AA, we performed an RNA-seq analysis on N2 (coated) and N2+AA (AA) cultured lt-NES cells. We found that 3050 genes were significantly upregulated and 1967 genes were downregulated after AA culture (Fig. 6a).

To understand the biological mechanism underlying the effects of AA, we performed a gene ontology enrichment analysis on differentially expressed genes. The 10 most significant categories were identified in the cell composition, molecular function, and biological process groups, respectively (Fig. 6b). The most remarkable categories were concentrated in the regulation of extracellular matrix (ECM) assembly, collagen metabolic process within biological process, and extracellular matrix component within cell composition (Fig. 6b). Among them, cell adhesion molecule binding was category that exhibited the greatest number of differences (Fig. 6c). The results reported above indicate that AA plays a critical role in cell adhesion, ECM deposition/remodeling and collagen synthesis, which is consistent with previous research [27, 28]. To understand the biological impact of these differentially expressed transcripts, next we performed a Kyoto Encyclopedia of Genes and Genomes functional analysis (Fig. 6d). The 20 significantly different pathways identified were mainly related to cell adhesion and ECM-receptor interaction.

Overall, these transcriptional profiling results indicate that AA is involved in the regulation of cell adhesion and is closely related to the regulation of ECM deposition/remodeling, especially in collagen synthesis.

### AA promotes the generation of lt-NES cells through the MEK-ERK1/2 pathway by promoting collagen synthesis

**Fig. 7:**
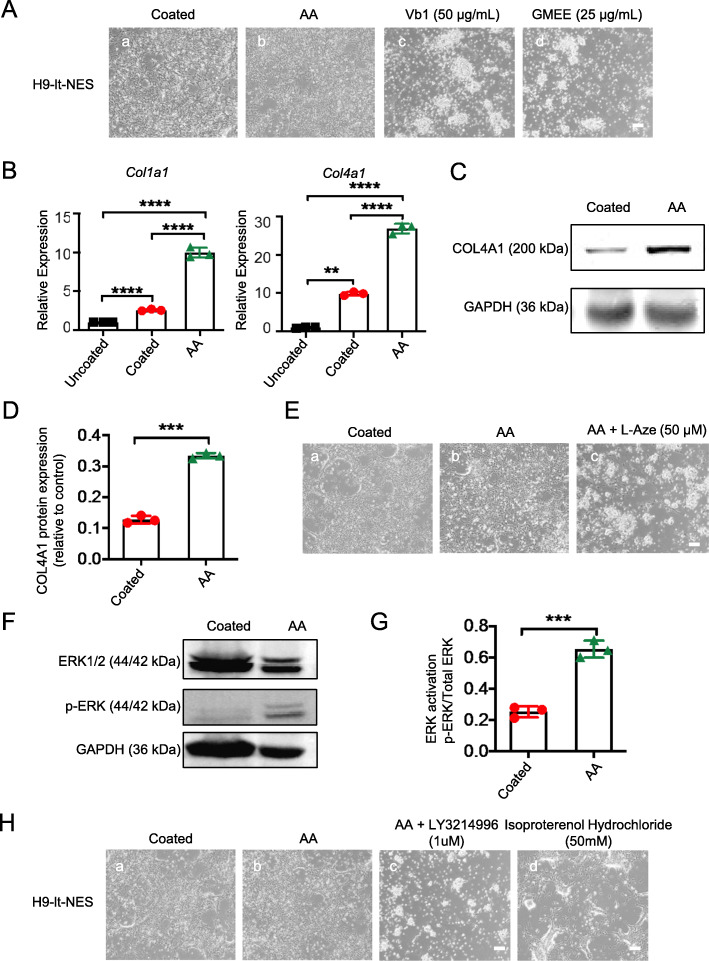
AA activates ERK signaling in a collagen synthesis-dependent manner. **a** The morphology of lt-NES cells treated with AA or alternative antioxidants vitamin B1 (Vb1) and reduced glutathione (GMEE). **b** Quantitative RT-PCR analysis of the relative expression of collagen genes *Col1a1* and *Col4a1*. *n* = 3 each. Data are expressed as means ± SD.*P < 0.05, **P < 0.01 vs. control. **c** Collagen deposition after AA treatment by Western blot analyze. **d** Quantitative statistics of collagen deposition after AA treatment by Western blot analyze. **e** The morphology of lt-NES cells treated with AA and collagen synthesis inhibitor AzC. **f** The whole-cell extracts of N2 cultured (coated) and N2+AA cultured (AA) lt-NES cells were analyzed by Western blot with phospho-specific antibodies against ERK1/2 (pERK1/2) and total ERK1/2. GAPDH was used as a control. **g** ERK activation is defined as the ratio of p-ERK/total ERK. *n* = 3 each. Data are expressed as means ± SD.*P < 0.05, **P < 0.01 vs. control. **h** The morphology of lt-NES cells when treated with or without ERK inhibitor and ERK activator. Scale bars = 100 μm

A transcriptome analysis indicated that AA plays a role by affecting cell adhesion, ECM deposition/remodeling, and collagen synthesis. However, it is known that AA is also an antioxidant; therefore, we aimed to determine whether the generation of lt-NES cells by AA can be attributed to its antioxidant properties. We found that treatment with other antioxidants, such as vitamin B1 (Vb1) and reduced glutathione (GMEE), did not mimic the effect of AA on lt-NES cell adhesion (Fig. 7A), indicating that the effect of AA of promoting the adherence of lt-NES cells was not related to its antioxidant properties. Collagens are indispensable components of the ECM, play a vital role in cell development and function, and have been shown to affect cell proliferation and differentiation [29]. In addition, it has been pointed out that AA enhanced the proliferation of CPCs via the MEK-ERK1/2 pathway through the manipulation of collagen synthesis [24]. To verify whether the mechanism underlying the promotion of the generation and expansion of lt-NES cells by AA was similar to that reported in the article mentioned above, we analyzed the effect of AA on collagen synthesis and found that the expression of the collagen genes *Col1a1* and *Col4a1* in lt-NES cells cultured in N2+AA (AA) was significantly increased (Fig. 7B), which is consistent with the transcriptome results. Moreover, western blot analysis further confirmed the strong increase in the expression of type IV collagen (Col IV), which demonstrated that AA can promote collagen deposition (Fig. 7C, D). To further explore the role of AA in the synthesis of collagen, we used the generic collagen synthesis inhibitor l-2-azetidine carboxylic acid (AzC) and found that the adhesion of It-NES cells afforded by AA was completely eliminated by AzC (50 μmol/l) (Fig. 7E). Furthermore, to confirm whether AA promotes collagen synthesis via the MEK-ERK1/2 pathway, we validated the AA-induced ERK1/2 activation and assessed the effect of ERK1/2 inhibitors. The western blot analysis shown in Fig. 7F, G indicated that the level of p-ERK1/2 was higher in N2+AA (AA) cultured lt-NES cells than it was in N2 cultured (coated) cells. Moreover, an ERK1/2 inhibitor (LY3214996) completely abolished the AA-induced generation and expansion of lt-NES cells (Fig. 7H), whereas the N2-cultured cells remained unchanged after the addition of LY3214996. Moreover, the use of isoproterenol hydrochloride as an activator of ERK partially mimicked the adhesion-promoting effect of AA on lt-NES cells, suggesting that the MEK-ERK1/2 pathway is involved in the AA-dependent generation and expansion of lt-NES cells.

The data reported above demonstrated that the AA-induced collagen synthesis promotes the generation and expansion of lt-NES cells through the MEK-ERK1/2 pathway.

## Discussion

In this study, a new system was established to induce lt-NES cells from hESC/hiPSC-neurospheres under chemically defined conditions, and this system was designed to meet the requirements of quality in clinical settings. This system also demonstrated that the N2+AA culture system is suitable for the induction and long-term expansion of lt-NES cells (Figs. 2, 3, and 4) and the differentiation of generic neurons (Fig. 5). The addition of AA to the N2 medium for the generation and expansion of It-NES cells allowed the elimination of pre-coating and the use of basement membrane preparations; therefore, the chemical composition of this culture system was more specific and this culture system complies with the standards of serum-free systems for clinical application in the future. Moreover, this system is suitable for the generation of various pluripotent stem cells, including ESCs and iPSCs; therefore, patient-specific iPSCs can be used to avoid ethical issues and immune rejection.

Neural stem/progenitor cells (NS/PCs) exhibit long-term expansion and constant self-renewal and have the ability to differentiate into a variety of neural cell lineages, including neurons and astrocytes, which illustrates the potential of neural stem cells in cell-replacement therapies for neurological disorders and injuries caused by the loss of neurons and glial cells, such as SCI [30, 31]. Neurospheres, a classic culture system for expanding NS/PCs, are cultivated as free-floating aggregates and are considered as a more natural environment for cells because of their 3D niche-like structure [15]. The methods used for neurosphere induction and expansion exhibit slight modifications from the previous protocols. Neurospheres are usually derived from single-cell suspensions of neural stem and progenitor cells isolated from the adult or fetal central nervous system (CNS); however, neurospheres can also be established from ES cells [32]. Here, we used a neural stem cell induction medium to induce hESCs/hiPSCs into rosettes, followed by rosette digestion and cell spreading onto uncoated plates in the form of a single-cell suspension. Subsequently, the single cells aggregated to form a spherical shape and expand continuously (Fig. 1). The medium was supplemented with B27 containing the fibroblast growth factor (bFGF) and EGF [33]. This neurosphere-induction method was proposed for the first time in this study and exhibited a higher yield and a higher efficiency than previous methods.

However, heterogeneity is one of the significant limitations of the neurosphere culture system [34]. This heterogeneity is caused by the size of the neurospheres, which is difficult to control and is usually not uniform; moreover, the cells inside and outside the neurospheres are exposed to different environments. This heterogeneity makes it difficult to maintain their long-term expansion and excellent differentiation potential. Moreover, after the transplantation of neurospheres into the nervous system, their neurogenic potential is gradually lost and the yield of neurons decreases [34]. Therefore, there is a need to develop a new NS/PC culture system that should be homogeneous, robust, and stable over time and that can constantly produce a large number of neurons. Culture of NSCs in a monolayer has been studied previously to address the above-mentioned needs, and human long-term self-renewing neuroepithelial-like stem cells (lt-NES cells) are NS/PCs grown in a monolayer culture system. They are a fairly homogeneous population of undifferentiated cells with the ability to continuously expand and stably produce functional neurons and glia [21]. The most common method used to generate lt-NES cells consists in the pre-coating of the culture plates with basement membrane preparations rich in extracellular matrix (ECM) components, such as laminin [35], fibronectin, gelatin [36], and Matrigel [37]. However, the basement membrane preparations listed above all contain exogenous components, which hinder the progress of cell transplantation because of uncertain chemical composition. Here, we established a new system for inducing and maintaining lt-NES cells under chemically defined conditions using AA (Fig. 2A). The lt-NES cells cultured under this new system can stably expand in large numbers and preserve their neural pluripotency (Figs. 2 and 3); moreover, they can maintain long-term culture (Fig. 4). Moreover, lt-NES cells cultured under our system can maintain the differentiation potential of functional neurons and glial cells (Fig. 5). Therefore, it is expected that this system will contribute to regenerative medicine for spinal cord injury and other CNS diseases in the future, because this method can reliably provide a large number of functional cells for regenerative medicine.

AA is widely known as an essential nutrient for guinea pigs and primates [38, 39]. AA has two major biological activities: it serves as an antioxidant and as a cofactor in collagen synthesis [40]. In our study, other antioxidants were tested, such as vitamin B1 (Vb1) and reduced glutathione (GMEE), but none of them mimicked the effect of AA on lt-NES cells (Fig. 7A), suggesting that antioxidant activity is not a critical factor in the generation and expansion of lt-NES cells. Therefore, we focused on the role of AA in the synthesis of collagen, as the ECM may affect cell adhesion. AA is essential for the biosynthesis of collagen. It is a cofactor of prolyl and lysyl hydroxylase and a stimulator of collagen gene expression. After the addition of AA to the culture medium, many studies have evaluated the relationship between AA and collagen expression regarding the short- and long-term effects on cells [27]. This study evaluated the use of AA in the long-term culture of It-NES cells. The results of the RNA-seq analysis (Fig. 6) revealed that AA affects cell acquisition and long-term culture by affecting cell adhesion, ECM remodeling, and collagen synthesis. Moreover, during the long-term culture, the expression of the collagen genes *Col1a1* and *Col4a1* was enhanced (Fig. 7B), and the adhesion of It-NES cells triggered by AA were completely eliminated by the collagen synthesis inhibitor AzC (50 μmol/l) (Fig. 7E). These data indicate that AA plays a promotive role in the generation and expansion of lt-NES cells because of collagen synthesis, rather than its antioxidant properties. It has been reported that AA specifically enhances CPC proliferation by manipulating collagen synthesis through the MEK-ERK1/2 pathway [24], which is consistent with our results, as the MEK-ERK1/2 pathway was activated by AA and the adhesion of It-NES cells induced by AA was completely abolished by an ERK inhibitor (1 μM) (Fig. 7D–F). We also treated the cells with an activator of ERK (isoproterenol hydrochloride (50 mM)) and found that it can promote the adhesion of some lt-NES cells; however, it cannot completely mimic the adhesion of AA to lt-NES cells. This may be because isoproterenol has other effects at the same time, and this result indicates that the MEK-ERK pathway is partly responsible for the promotion of lt-NES adhesion by AA; in addition, other signaling pathways may be involved in this process. Moreover, we found that lt-NES cells cultured in N2+AA (AA) had higher expression levels of NS/PC markers than did cells cultured in N2 (coated) (Figs.2 and 3). The studies mentioned above indicate that AA may play a critical role in the development of NS/PCs and may provide a more suitable culture environment for lt-NES cells and promote the neural pluripotency of lt-NES cells.

## Conclusion

We have successfully established a new culture system for the long-term large-scale monolayer culture of NS/PCs from hESC/hiPSCs. AA promoted the formation of a homogenous population of lt-NES cells grown in an environment without foreign components. After 10–15 passages, the cells on the N2+AA condition can maintain good self-renewal ability, and the cells can retain neural pluripotency and the ability to differentiate into generic functional neurons. In addition, we demonstrated that AA specifically promotes the generation and expansion of neuroepithelial-like stem cells through the MEK-ERK1/2 pathway by increasing collagen synthesis. The findings of this study will help promote the clinical application of hPSC-derived NS/PCs in regenerative medicine for SCI and neurological diseases.

## Supplementary Information

**Additional file 1: Supplementary Table S1.** Primer sequences used for q-PCR.

**Additional file 2: Figure S1.** The morphologies of the lt-NES cells in different medium. (A) The morphologies of the lt-NES cells in N2 medium (coated), N2+AA (AA), and N2+ Retinoic acid, Y27632,Tzv and Blebbiststin. Scale bars = 100 μm. **Figure S2.** Long-term growth curves of lt-NES cells. (A) The cumulative population doublings (CPD) of lt-NES cells cultured by coated and AA. Every cell passage is indicated by a point and thenumber of CPD was calculated based on the ratio of cells seeded versus cells harvested per passage. **Figure S3.** lt-NES cells can differentiated into astrocyte and oligodendrocyte .(A) Quantitative RT-PCR analysis of the expression level of the astrocyte marker GFAP and the oligodendrocyte marker OLIG2, and neurons markers NeuN and Neurog,*n* = 3 each.

## Data Availability

All data generated or analyzed during this study are included in this published article.

## Abbreviations

SCI: Spinal cord injury
hPSCs: Human pluripotent stem cells
NS/PCs: Neural stem/progenitor cells
AA: Ascorbic acid
lt-NES cells: Long-term self-renewing neuroepithelial-like stem cells
RT-PCR: Reverse transcription-polymerase chain reaction
MEA: Microelectrode array
mESCs: Mouse embryonic stem cells
hESCs: Human embryonic stem cells
iPSCs: Induced pluripotent stem cells
UiPSs: Urine-iPSCs
GFAP: Glial fibrillary acidic protein
ECM: Extracellular matrix
GO: Gene ontology
KEGG: Kyoto Encyclopedia of Genes and Genomes
Vb1: Vitamin B1
GMEE: Reduced glutathione
AzC: L-2-Azetidine carboxylic acid
CNS: Central nervous system
bFGF: Fibroblast growth factor
EGF: Epidermal growth factor
