# Supplementary material for: Ascorbic acid can promote the generation and expansion of neuroepithelial-like stem cells derived from hiPS/ES cells under chemically defined conditions through promoting collagen synthesis
Source: Stem Cell Res Ther. 2021 Jan 9;12:48. doi: 10.1186/s13287-020-02115-6 (PMC7796386; doi:10.1186/s13287-020-02115-6)
Supplement: Supplementary file 1 — Additional file 1: Supplementary Table S1. Primer sequences used for q-PCR. [file 13287_2020_2115_MOESM1_ESM.docx]

**Supplementary**

Table S1. Primer sequences used for q-PCR

| Gene | Forward 5’-3’ | Reverse 3’-5’ |
| --- | --- | --- |
| PAX6 | CTGAGGAATCAGAGAAGACAGGC | ATGGAGCCAGATGTGAAGGAGG |
| NESTIN | TCAAGATGTCCCTCAGCCTGGA | AAGCTGAGGGAAGTCTTGGAGC |
| SOX1 | GAGTGGAAGGTCATGTCCGAGG | CCTTCTTGAGCAGCGTCTTGGT |
| SOX2 | GCTACAGCATGATGCAGGACCA | TCTGCGAGCTGGTCATGGAGTT |
| OCT4 | CCTGAAGCAGAAGAGGATCACC | AAAGCGGCAGATGGTCGTTTGG |
| NANOG | CTCCAACATCCTGAACCTCAGC | CGTCACACCATTGCTATTCTTCG |
| NeuN | TACGCAGCCTACAGATACGCTC | TGGTTCCAATGCTGTAGGTCGC |
| TUBB3 | TCAGCGTCTACTACAACGAGGC | GCCTGAAGAGATGTCCAAAGGC |
| NEUROG | CAAGCTCACCAAGATCGAGACC | AGCAACACTGCCTCGGAGAAGA |
| vGlut1 | GCAAGTACATCGAGGACGCCAT | GCCACGATGATGGCATAGACTG |
| GFAP | CTGGAGAGGAAGATTGAGTCGC | ACGTCAAGCTCCACATGGACCT |
| S100B | ACGTCAAGCTCCACATGGACCT | TCCTGGAAGTCACATTCGCCGT |
| Col1a1 | GATTCCCTGGACCTAAAGGTGC | AGCCTCTCCATCTTTGCCAGCA |
| Col4a1 | TGTTGACGGCTTACCTGGAGAC | GGTAGACCAACTCCAGGCTCTC |
| GAPDH | GGAGCGAGATCCCTCCAAAAT | GGCTGTTGTCATACTTCTCATGG |
