# Supplementary material for: Ascorbic acid can promote the generation and expansion of neuroepithelial-like stem cells derived from hiPS/ES cells under chemically defined conditions through promoting collagen synthesis
Source: Stem Cell Res Ther. 2021 Jan 9;12:48. doi: 10.1186/s13287-020-02115-6 (PMC7796386; doi:10.1186/s13287-020-02115-6)
Supplement: Supplementary file 2 — Additional file 2: Figure S1. The morphologies of the lt-NES cells in different medium. (A) The morphologies of the lt-NES cells in N2 medium (coated), N2+AA (AA), and N2+ Retinoic acid, Y27632,Tzv and Blebbiststin. Scale bars = 100 μm. Figure S2. Long-term growth curves of lt-NES cells. (A) The cumulative population doublings (CPD) of lt-NES cells cultured by coated and AA. Every cell passage is indicated by a point and thenumber of CPD was calculated based on the ratio of cells seeded versus cells harvested per passage. Figure S3. lt-NES cells can differentiated into astrocyte and oligodendrocyte .(A) Quantitative RT-PCR analysis of the expression level of the astrocyte marker GFAP and the oligodendrocyte marker OLIG2, and neurons markers NeuN and Neurog,n = 3 each. [file 13287_2020_2115_MOESM2_ESM.pdf]

Figure S1.

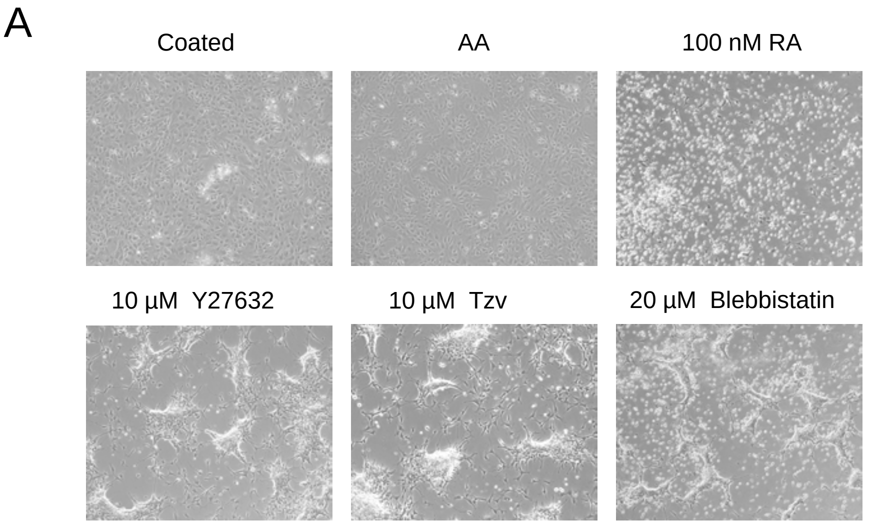

Figure S1. The morphologies of the lt-NES cells in different medium

(A) The morphologies of the lt-NES cells in N2 medium (coated) , N2+AA (AA), and N2+ Retinoic acid, Y27632,Tzv and Blebbiststin. Scale bars = 100 µm

Figure S2.

A

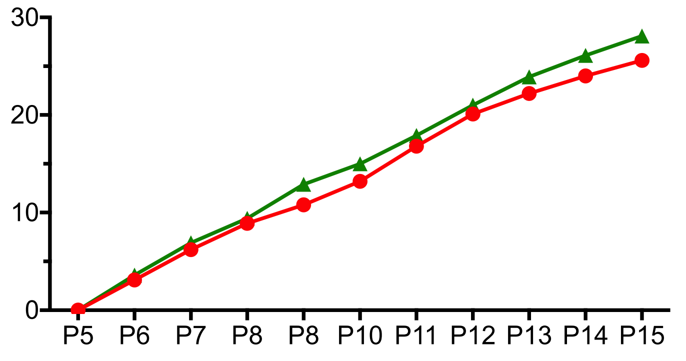

Figure S2. Long - term growth curves of lt-NES cells .

(A)The cumulative population doublings (CPD) of lt-NES cells cultured by coated and AA. Every cell passage is indicated by a point and thenumber of CPD was calculated based on the ratio of cells seeded versus cells harvested per passage.

Figure S3.

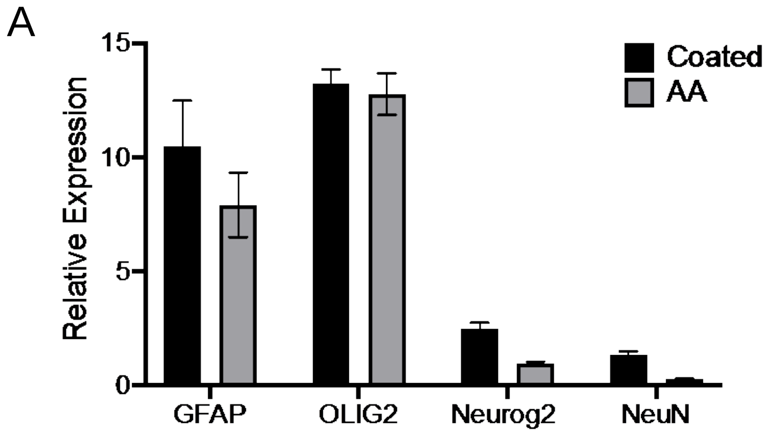

Figure S3. lt-NES cells can differentiated into astrocyte and oligodendrocyte .

(A) Quantitative RT-PCR analysis of the expression level of the astrocyte marker GFAP and the oligodendrocyte marker OLIG2, and neurons markers NeuN and Neurog,,n = 3 each.
